# An Integrated Approach of Proteomics and Computational Genetic Modification Effectiveness Analysis to Uncover the Mechanisms of Flood Tolerance in Soybeans

**DOI:** 10.3390/ijms19051301

**Published:** 2018-04-26

**Authors:** Xin Wang, Katsumi Sakata, Setsuko Komatsu

**Affiliations:** 1 Graduate School of Life and Environmental Sciences, University of Tsukuba, Tsukuba 305-8572, Japan; wangxin@affrc.go.jp; 2 National Institute of Crop Science, National Agriculture and Food Research Organization, Tsukuba 305-8518, Japan; 3 Department of Life Science and Informatics, Maebashi Institute of Technology, Maebashi 371-0816, Japan

**Keywords:** flooding tolerance, proteomics, genetic modification-effectiveness analysis, abscisic acid, mutant, soybean

## Abstract

Flooding negatively affects the growth of soybeans. Recently, omic approaches have been used to study abiotic stress responses in plants. To explore flood-tolerant genes in soybeans, an integrated approach of proteomics and computational genetic modification effectiveness analysis was applied to the soybean (*Glycine max* L. (Merrill)). Flood-tolerant mutant and abscisic acid (ABA)-treated soybean plants were used as the flood-tolerant materials. Among the primary metabolism, glycolysis, fermentation, and tricarboxylic acid cycle were markedly affected under flooding. Fifteen proteins, which were related to the affected processes, displayed similar protein profiles in the mutant and ABA-treated soybean plants. Protein levels of glyceraldehyde-3-phosphate dehydrogenase (GAPDH), aconitase 1, and 2-oxoglutarate dehydrogenase were higher in flood-tolerant materials than in wild-type soybean plants under flood conditions. These three proteins were positioned in each of the three enzyme groups revealed by our computational genetic modification effectiveness analysis, and the three proteins configured a candidate set of genes to promote flood tolerance. Additionally, transcript levels of GAPDH were similar in flood-tolerant materials and in unstressed plants. These results suggest that proteins related to energy metabolism might play an essential role to confer flood tolerance in soybeans.

## 1. Introduction

Soybeans are an essential crop that is not only rich in protein and vegetable oil but also rich in isoflavones and phenolic compounds [1,2]. Although soybeans are grown in a wide range of environmental conditions, plant growth and grain yield are markedly affected by unfavorable conditions, such as flooding [3]. Energy management is affected in a flooded soybean in terms of its biotin, biotinylation [4], and calcium homeostasis [5]. Protein synthesis and glycoprotein folding are suppressed in flooded soybeans [6]. In addition, *S*-adenosylmethionine synthetase is induced by flooding [7], which is associated with ethylene biosynthesis [8]. These findings indicate that a series of processes are involved in the overall flooding response in soybeans.

In soybean seedlings, flooding decreases both the weight and length of roots, including the hypocotyl, leading to growth suppression [9]. Genes associated with alcoholic fermentation, ethylene biosynthesis, cell wall loosening, and pathogen defense are upregulated in soybean roots, including the hypocotyl, when flooded [10]. In the post-flooding recovery of soybean seedlings, both alteration of cell structure [11] and scavenging of toxic radicals [12] have been shown to play important roles. These findings indicate that the soybean root, including the hypocotyl, is susceptible to flooding. It is necessary to reveal the specific mechanisms of the soybean which respond to flooding; such mechanisms will provide insights into developing flood tolerance within the plant.

The flood-tolerant soybean, which was generated using gamma-ray irradiation and coupled with flood-tolerant tests, survived after seven days of stress and its root tip was not affected by flooding conditions [13]. When exposed to flooding, the survival ratio of a soybean was improved when plant growth was controlled by additional abscisic acid (ABA) treatment. The sugar metabolism of the soybean also contributed to flood tolerance via the regulation of zinc finger proteins, cell division cycle 5 protein, and transducin, which were induced by ABA [14]. Subsequently, it was determined that the metabolic processes of protein synthesis and RNA regulation contributed to triggering tolerance to initial flooding stress in the flood-tolerant soybean and ABA-treated plant; concurrently, the integrity of cell wall and balance of glycolysis were key factors for the survival stages [15]. These results support the conclusion that ABA treatment to flooded soybeans exhibited tolerant characteristics such as an increased survival ratio; it also suggests that the flooded soybean with ABA treatment as well as the flood-tolerant soybean could provide the materials to investigate flood tolerance mechanisms of the soybean.

Omic techniques have facilitated the development of stress-tolerant crops [16]. Genes, proteins, and metabolites, which were related to flooding-response mechanisms in soybeans, were integrated, indicating that both the activation of glycolysis and fermentation were key elements [17]. Moreover, changes to genes, proteins, and metabolites in soybeans over time highlighted the significant relationship among these different omics [18]. In addition, a kinetic model of the metabolism provided information on regulatory mechanisms and displayed dynamic behavior in bacteria [19]. Recently, kinetic models, combined with transcriptomic, proteomic, and metabolomic data, described the applications of the numerous variations of state in plants or animals under external stimuli [20]. Mathematical models were constructed based on metabolites reported by Nakamura et al. [21] and metabolic profiles were simulated under flood conditions, indicating a loss of variation of state during the flooding response of soybeans [20]. These findings indicate that a kinetic model integrated with omic analyses could be utilized to explore stress responses in plants.

To explore the mechanisms of flood tolerance in soybeans, a metabolic simulation integrated with proteomic analysis was applied. Wild-type soybeans treated with ABA and flood-tolerant mutants were used as the flood-tolerant materials. Proteins were identified using a gel-free/label-free proteomic technique. The response of metabolism to flood conditions were examined and proteins which displayed the same levels as flood-tolerant materials were subjected to metabolic simulation. On the basis of the reduction ratios which were calculated by the simulation, proteins related to flood tolerance were identified. An integrated data set, including gene expression and protein abundance, is beneficial to reveal regulatory mechanisms on multiple levels [22]. In this study, gene expression was examined and integrated with proteomic data to validate the results of metabolic simulation. The present study will facilitate the development of a flood-tolerant soybean through various attempts to genetically modify potential candidates, which were selected by simulation and displayed correspondence with gene expression and protein abundance.

## 2. Results

### 2.1. Effects of Flooding on Soybean Morphology

**Figure 1:**
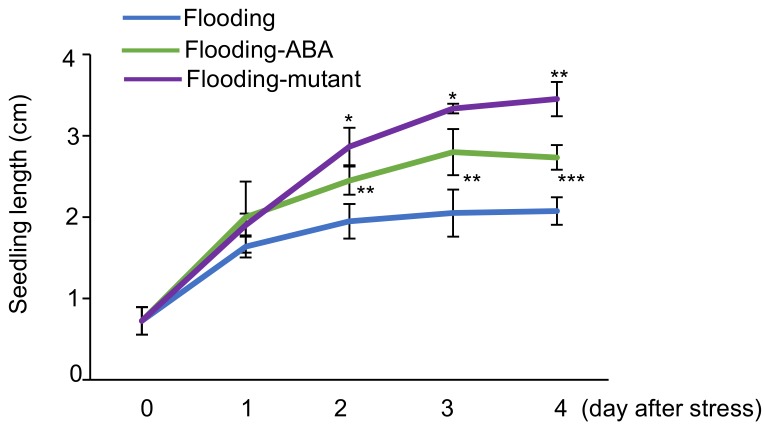
Seedling length of wild-type soybean, abscisic acid (ABA)-treated soybean, and flood-tolerant mutant soybean plants under flooding. Two-day-old wild-type soybean and flood-tolerant mutant soybean plants were exposed to flooding. For ABA treatment, 10 μM ABA was supplied to wild-type soybean plants exposed to flooding at the same time. The seedling length, which is the length of root including the hypocotyl, was measured at each time point. Data are mean ± standard deviation (SD) from three independent biological replications. Flooded wild-type soybean plants at each time point were used for comparison. * *p* < 0.05, ** *p* < 0.01, and *** *p* < 0.001 (Student’s *t*-test).

To examine the effects of flooding on soybean growth, seedling length, which is the root including the hypocotyl, was measured. Two-day-old soybean plants were exposed to flooding for 1, 2, 3, and 4 days and images were taken at each time point (Figure S1). The seedling length was measured at indicated time points (Figure S1). The seedling length of flood-tolerant materials, which included ABA-treated wild-type soybean and flood-tolerant mutant plants, was compared with wild-type soybean under flooding conditions. The seedling length was shorter in flooded soybeans compared with unstressed plants (Figure S2); however, it was longer in flood-tolerant materials than in wild-type soybean under flooding conditions (Figure 1). These results indicated that flooding adversely affected soybean growth and that flood-tolerant materials grew better than wild-type soybean under flooding conditions. Because morphological changes were visible and measurable during one to four days of flooding, this duration of flooding stress was used in the following experiments to elucidate the flood-tolerant mechanisms of the soybean.

### 2.2. Effects of Flooding on the Primary Metabolism in Soybeans

**Figure 2:**
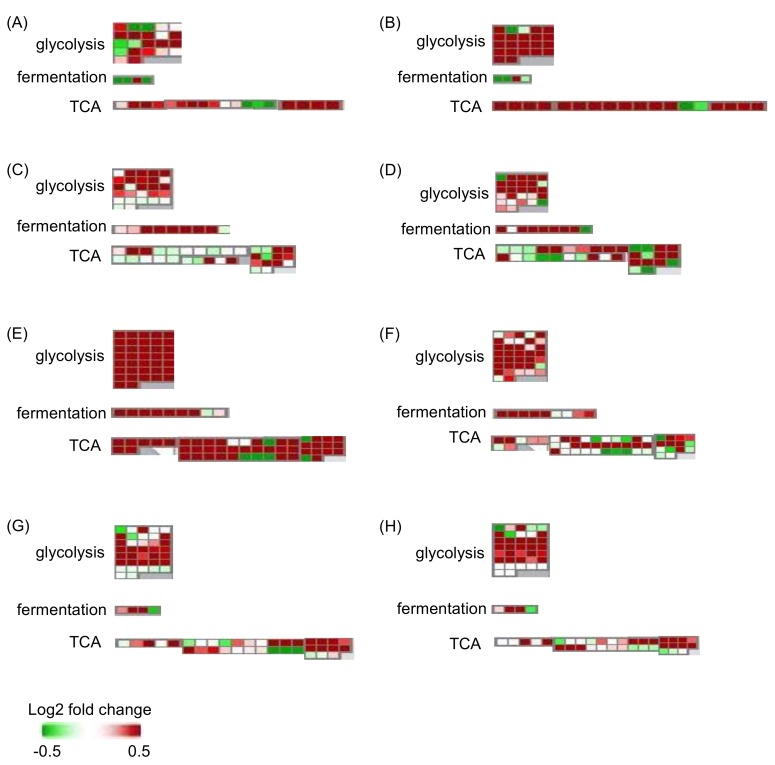
Abundance of proteins mapped to glycolysis, fermentation, and tricarboxylic acid cycle in soybean plants. (**A**) Four-day-old wild-type soybean without flooding stress. (**B**) Six-day-old wild-type soybean without flooding stress. (**C**) Four-day-old wild-type soybean flooded for two days. (**D**) Six-day-old wild-type soybean flooded for four days. (**E**) Four-day-old wild-type soybean flooded for two days coupled with ABA treatment. (**F**) Six-day-old wild-type soybean flooded for four days coupled with ABA treatment. (**G**) Four-day-old flooding-tolerant soybean flooded for two days. (**H**) Six-day-old flooding-tolerant soybean flooded for four days. Two-day-old wild-type soybean plants were treated without or with flooding for two and four days. For ABA treatment, 10 μM ABA was supplied to wild-type soybean plants exposed to flooding at the same time. Two-day-old flood-tolerant mutant plants were exposed to flooding for two and four days. Unstressed wild-type soybean plants served as control. Green and red colors indicate a decrease and increase, respectively, in fold change values compared with two-day-old unstressed wild-type soybean plants. Abbreviation: TCA, tricarboxylic acid cycle.

To explore the flood-tolerant mechanisms of soybeans, a gel-free/label-free proteomic technique was performed (Figure S1). Two-day-old wild-type soybean plants were treated with or without flooding, and ABA was supplied to the flooded plants at the same time. Two-day-old flood-tolerant mutant plants were subjected to flooding. Samples of root (including the hypocotyl) were collected at indicated time points. Proteins were extracted and analyzed under different conditions (Tables S1–S4). Fold change of protein level was visualized using MapMan software (Figures S3 and S4). Among the primary metabolism, glycolysis, fermentation, and tricarboxylic acid cycle were predominantly affected by flooding conditions (Figure 2). Glycolysis and tricarboxylic acid cycle were enhanced in unstressed plants compared with flooded ones. Compared with unstressed plants, flooded wild-type plants displayed suppressed glycolysis and tricarboxylic acid cycle, but enhanced fermentation. Glycolysis and tricarboxylic acid cycle were enhanced in ABA-treated soybean and flood-tolerant mutant plants, compared with flooded wild-type soybeans. Tricarboxylic acid cycle was slightly suppressed in flood-tolerant materials compared with unstressed plants (Figure 2).

### 2.3. Genetic Modification Effectiveness Analysis Related to Glycolysis, Fermentation, and Tricarboxylic Acid Cycle in Soybeans

**Figure 3:**
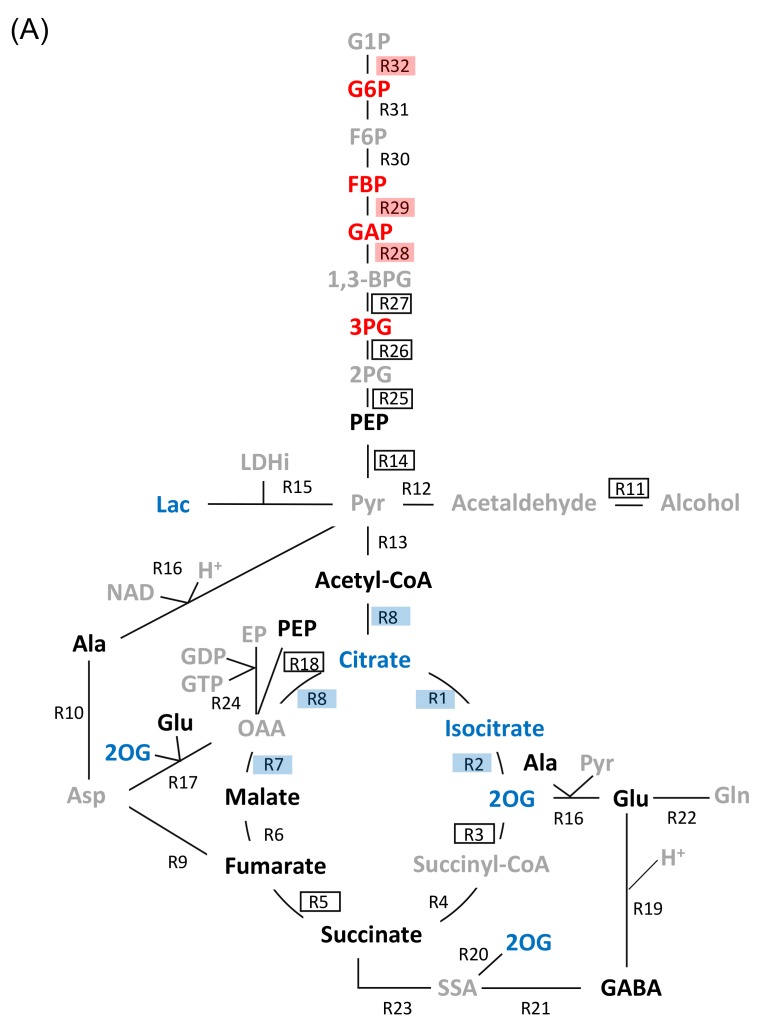

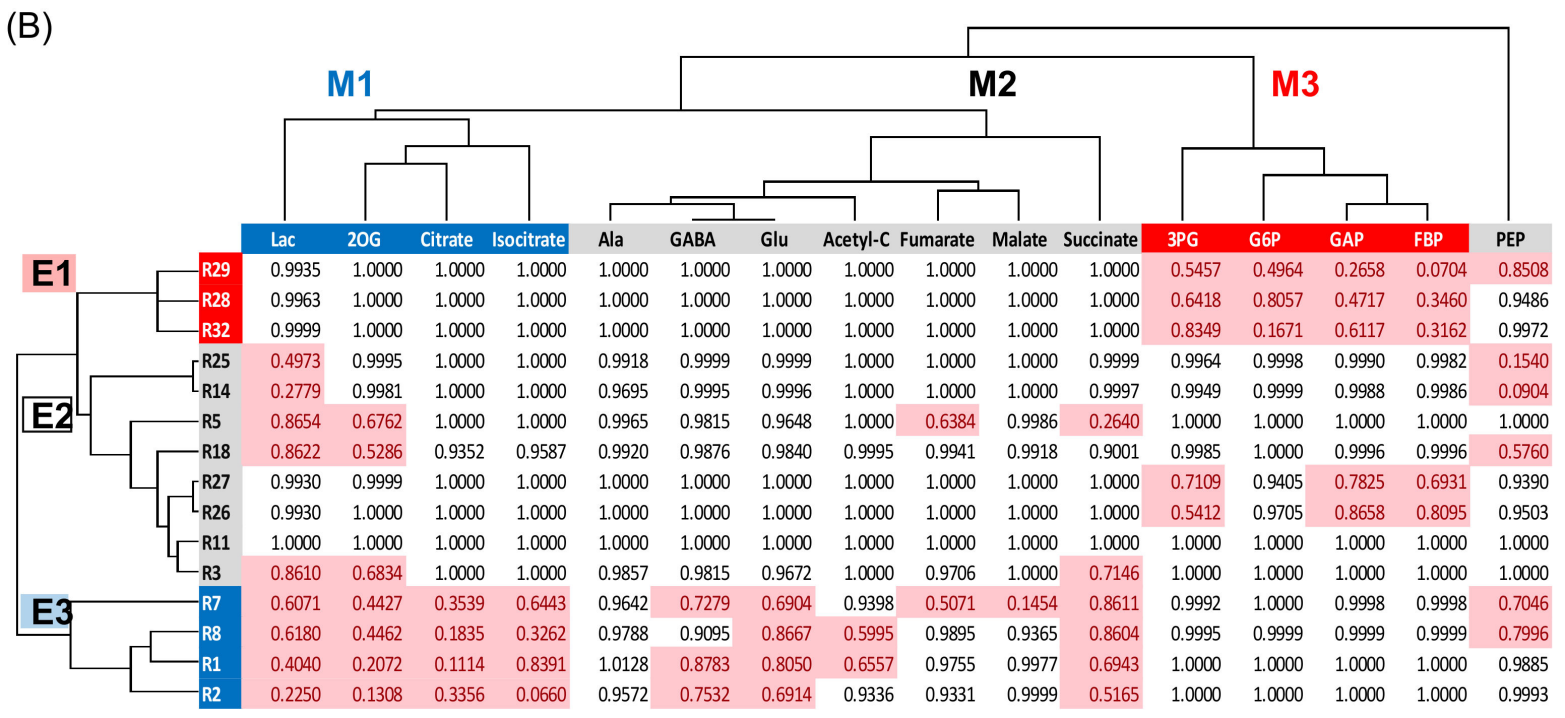
Soybean metabolic model and genetic modification effectiveness analysis. (**A**) Schematic diagram of soybean metabolic model. “Rx” indicates reaction number (Table S5). Sixteen target-fitting metabolites presented with blue, black, and red colors indicate the metabolites in M1, M2, or M3 groups, respectively. Metabolites presented with gray color are included in model but not target-fitting metabolites. The “Rx” circled with red, black, and blue colors indicate the enzymes in E1, E2, and E3 groups, respectively. (**B**) Result of genetic modification effectiveness analysis. Clustering was conducted on the basis of metabolite accumulation reduction ratio. “E” and “M” indicate the enzyme and metabolite, respectively. Tabulated reduction ratios were subjected to hierarchical clustering analysis, which was a centroid linkage method based on a Euclidean distance metric. Metabolites presented with blue, gray, and red colors are those divided into M1, M2, and M3 groups, respectively. Red cell in the table indicates accumulation reduction ratio, which was lower than 0.9 for corresponding enzyme modification-metabolite pair. Abbreviations are as follows: 1,3-BPG, 1,3-bisphosphoglyceric acid; 2OG, 2-oxoglutarate; 2PG, 2-phosphoglyceric acid; 3PG, 3-phosphoglyceric acid; Acetyl-CoA, acetyl-coenzyme A; Ala, alanine; Asp, aspartic acid; EP, enolpyruvate; F6P, fructose 6-phosphate; FBP, fructose bisphosphate; G1P, glucose-1-phosphate; G6P, glucose 6-phosphate; GABA, gamma-aminobutyric acid; GAP, glyceraldehyde-3-phosphate; GDP, guanosine 5’-diphosphate; Gln, glutamine; Glu, glutamate; GTP, guanosine 5’-triphosphate; Lac, lactate; LDHi, lactate dehydrogenase inhibitor; NAD, nicotinamide adenine dinucleotide; OAA, oxaloacetate; PEP, phosphoenol pyruvate; Pyr, pyruvate; SSA, succinic semialdehyde.

Glycolysis, fermentation, and tricarboxylic acid cycle were predominantly affected by flooding conditions in the soybeans (Figure 2). Proteins related to these metabolisms were divided into 15 biochemical reactions (Table 1 and Table 2) based on the model constructed by Sakata et al. [20]. It indicated that eight, one, and six biochemical reaction(s) were presented in glycolysis, fermentation, and tricarboxylic acid cycle, respectively (Figure 3A). Calculation for 15 enzyme reactions was conducted and, after adjusting the *V*_max_ values of these proteins, simulated amounts of 16 target-fitting metabolites (Figure 3B) were fitted to the experimental metabolic data of flooded soybeans reported by Nakamura et al. [21]. The log-transformed calculated data and experimental data of the target-fitting metabolites were plotted against each other (Figure S5).

We conducted a genetic modification effectiveness analysis to investigate the change of accumulation tendency of each metabolite that was caused by a modification of each enzyme protein (over or under expression with same fold-change value for each protein). The metabolite accumulation reduction ratios, which were calculated such that the virtual modifications of *V*_max_ values (4 or 0.25 × initial *V*_max_ value) were applied to the reactions models, and the calculation results were subjected to clustering analysis. The reduction ratio was calculated for both modifications (4 or 0.25 × initial *V*_max_ value) and the data of smaller reduction ratio by overexpression or suppression was selected (Figure 3B).

Results indicated that the enzymes were divided into E1, E2, and E3 groups, and metabolites were divided into M1, M2, and M3 groups, as well as phosphoenol pyruvate (Figure 3B). Moreover, an exclusive effect on reducing metabolite accumulation of M1, M2, and M3 groups was detected in the enzyme groups E1 and E3. The enzymes in the E1 group exclusively reduced the accumulation of metabolites in the M3 group. Enzymes in the E3 group exclusively reduced the accumulation of metabolites in the M1 and M2 groups. Furthermore, the corresponding effective enzyme-metabolite group pairs, in which an enzyme in the enzyme group effectively reduced metabolite accumulation in the metabolite group, were closely positioned in the metabolic network (Figure 3). It showed that enzymes in E1 group (red) were near metabolites in M3 group (red), enzymes in E2 group (black circled) were near metabolites in M2 group (black), and enzymes in E3 group (blue) were near metabolites in M1 group (blue), indicating that the group pairs were positioned close together in the metabolic network. On the basis of these observations, a strategy to configure a candidate gene set for flood-tolerant soybeans was suggested to collect effective enzymes from E1, E2, and E3 groups.

### 2.4. Cluster Analysis of Proteins Related to Glycolysis, Fermentation, and Tricarboxylic Acid Cycle in Soybeans

**Figure 4:**
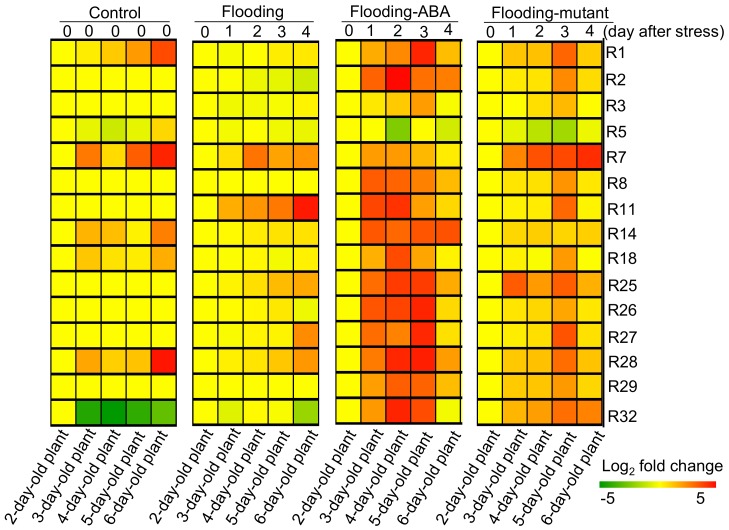
Cluster analysis of proteins related to glycolysis, fermentation, and tricarboxylic acid cycle in soybeans under flooding. Two-day-old wild-type soybean plants were treated without or with flooding for 1, 2, 3, and 4 days. For ABA treatment, 10 μM ABA was supplied to wild-type soybean plants exposed to flooding at the same time. Two-day-old flood-tolerant mutant plants were exposed to flooding for 1, 2, 3, and 4 days. Unstressed wild-type soybean plants served as controls. Roots samples (including the hypocotyl) were collected for protein extraction. SIEVE analysis was performed to examine protein abundance. Proteins related to glycolysis, fermentation, and tricarboxylic acid cycle were divided into 15 reactions (Table 1 and Table 2). They were subjected to cluster analysis based on protein abundance. Green and red colors indicate a decrease and increase, respectively, in protein abundance compared with two-day-old unstressed wild-type soybean plants.

Proteins related to glycolysis, fermentation, and tricarboxylic acid cycle (Table 1 and Table 2) were subjected to cluster analysis to screen the flood-tolerant candidates (Figure 4). The cluster analysis indicated that protein levels of R1 and R28 were increased in unstressed wild-type soybeans and in flood-tolerant materials under flooding conditions in a time-dependent manner. Protein levels of R3 decreased in flooded wild-type soybeans compared with unstressed soybeans but increased in flood-tolerant materials under flooding conditions (Figure 4). These results indicated that R1, R28, and R3 presented similar tendencies towards protein level changes between flood-tolerant materials and unstressed wild-type soybeans. We calculated the average time of expression level of each protein for the unstressed wild-type soybean (indicated as U), the wild-type soybean under flooding conditions (F), the ABA-treated soybean under flooding conditions (A), and the flood-tolerant mutant under flooding conditions (M). The ratios between the time average (U/F, A/F, M/F) were observed for protein R1 (3.11, 6.69, 3.23), R2 (0.76, 8.94, 1.68), R3 (1.1, 2.22, 1.67), R5 (0.66, 0.91, 0.7), R7 (1.89, 0.81, 2.60), R8 (0.75, 4.63, 1.57), R11 (0.85, 7.07, 2.39), R14 (1.91, 6.64, 1.36), R18 (1.84, 4.98, 1.85), R25 (0.53, 4.97, 3.15), R26 (0.92, 9.93, 1.76), R27 (0.63, 5.14, 2.28), R28 (2.73, 6.61, 1.81), R29 (1, 5.61, 2.39), and R32 (0.36, 3.18, 1.48). The ratios U/F, A/F, and M/F for protein R1, R28, and R3 are similar such as U/F > 1, A/F > 1 and M/F > 1; accordingly, a similar change tendency of protein levels among R1, R28, and R3 was observed. R1, R28, and R3 were positioned in the enzyme group E3, E1, and E2, respectively. The three proteins could configure the candidate gene set as a result of the strategy described in the previous Section 2.3.

**Figure 5:**
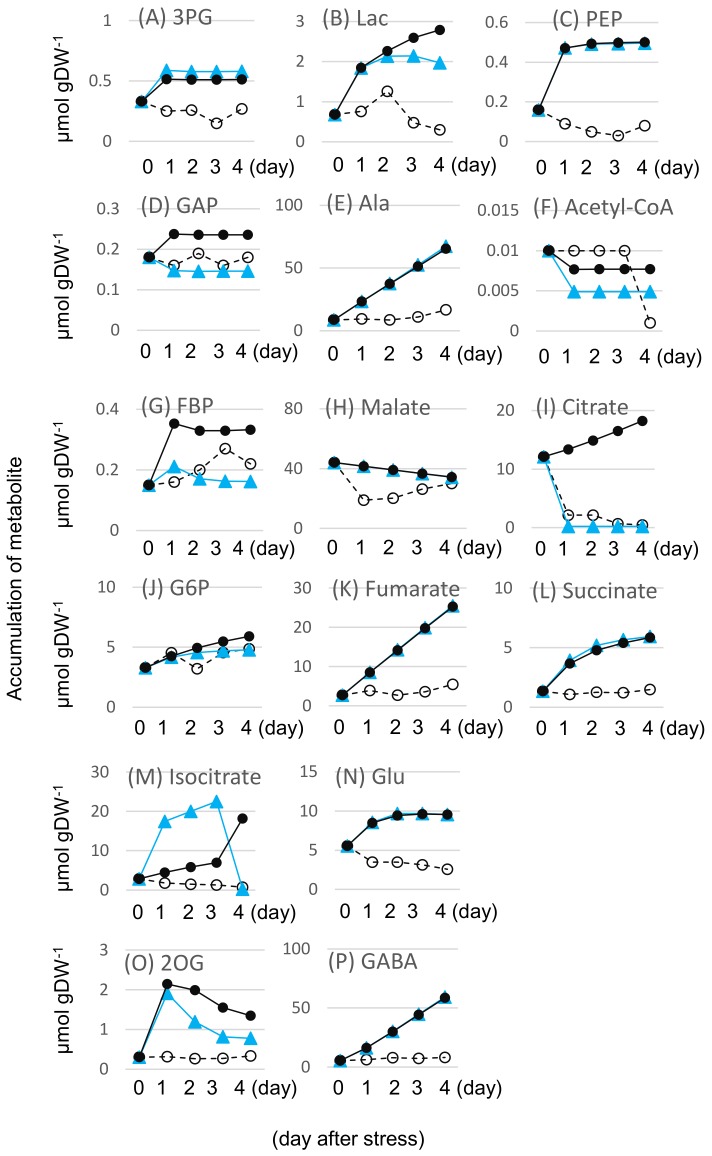
Temporal profiles of 16 metabolites for unstressed and flooding conditions with and without enzyme modification: (**A**) 3PG; (**B**) Lac; (**C**) PEP; (**D**) GAP; (**E**) Ala; (**F**) Acetyl-CoA; (**G**) FBP; (**H**) malate; (**I**) citrate; (**J**) G6P; (**K**) fumarate; (**L**) succinate; (**M**) isocitrate; (**N**) Glu; (**O**) 2OG; (**P**) GABA. In the enzyme modification, overexpression was mimicked by increasing for R1, R28, and R3, 3.2, 1.8, and 1.6×, respectively, the *V*_max_ value from its initial value (Table S5). Filled circle and blue triangle indicate simulated profiles under flooding without and with enzyme modification, respectively. Open circle indicates experimental profiles in unstressed conditions. The area below each temporal profile under flooding without and with enzyme modification was measured and was used to calculate metabolite accumulation reduction ratio. Horizontal and vertical axes indicate day-after stress and accumulation of metabolite, respectively. “DW” indicates dry weight of plant. The abbreviations of metabolites are the same as described in the legend of Figure 3.

Metabolic simulation was conducted by concurrent modification of R1, R28, and R3. In the simulation, *V*_max_ values of the three proteins were increased to mimic the overexpression such that 3.2, 1.8, and 1.6 × initial *V*_max_ value of R1, R28, and R3, respectively. Accordingly, the fold 3.2, 1.8, and 1.6 was the same as the observed ratios between the time average (M/F), indicating that the average time of the flood-tolerant mutant under flooding conditions compared to the wild-type soybean under flooding conditions. Temporal profiles of metabolites under the concurrent modifications of R1, R28, and R3 are shown in Figure 5. Out of 16 metabolites, the accumulation of seven metabolites was reduced. The reduced metabolites were lactate, 2-oxoglutarate, and citrate in M1 group; acetyl-coenzyme A in M2 group; and glucose 6-phosphate, glyceraldehyde-3-phosphate, and fructose bisphosphate in M3 group (Figure 5). The average accumulation reduction ratio of 0.933 was obtained.

### 2.5. Integration of Protein Level and Transcript Level

In the current study, the predominantly affected metabolisms under flooding conditions were examined using MapMan software (Figure 2). Fifteen biochemical reactions which were involved in the affected processes displayed similar protein profiles in the mutant and ABA-treated soybean plants (Table 2). Protein levels of 15 enzymes were examined and R1, R28, and R3 displayed similar change tendencies in flood-tolerant materials as in unstressed soybean plants in a time-dependent manner (Figure 4). The modifications of R1, R28, and R3 were further investigated using metabolic simulation.

**Figure 6:**
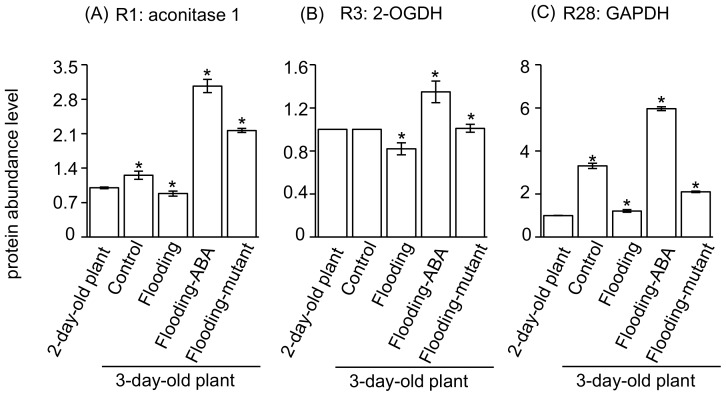
Protein abundance of aconitase 1,2-oxoglutarate dehydrogenase, and glyceraldehyde-3-phosphate dehydrogenase in soybean at an early stage of flooding. (**A**) Protein abundance of aconitase 1 (R1) in soybean at an early stage of flooding; (**B**) Protein abundance of 2-oxoglutarate dehydrogenase (R3) in soybean at an early stage of flooding; (**C**) Protein abundance of glyceraldehyde-3-phosphate dehydrogenase (R28) in soybean at an early stage of flooding. Two-day-old wild-type soybean plants were treated without or with flooding for one day. For ABA treatment, 10 μM ABA was supplied to wild-type soybeans exposed to flooding at the same time. Two-day-old flood-tolerant mutant plants were exposed to flooding for one day. Unstressed wild-type soybean plants served as controls. Root samples (including the hypocotyl) were collected for protein extraction. SIEVE analysis was performed to examine protein abundance. Data shown are means ± SD from three independent biological replications. Two-day-old unstressed wild-type soybean plants were used for comparison. * *p* < 0.05 (Student’s *t*-test). Abbreviations are as follows: 2-OGDH, 2-oxoglutarate dehydrogenase; GAPDH, glyceraldehyde-3-phosphate dehydrogenase.

**Figure 7:**
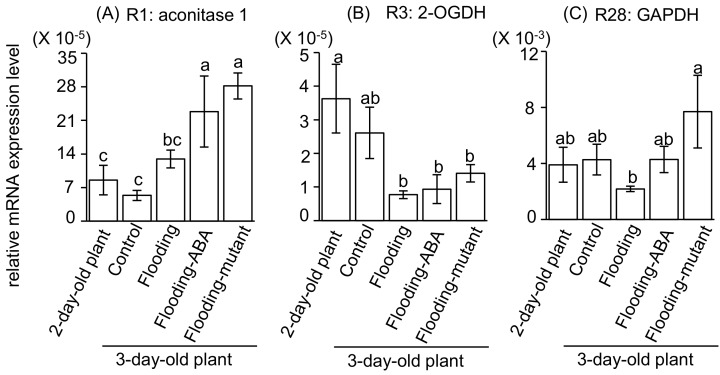
Gene expression of aconitase 1,2-oxoglutarate dehydrogenase, and glyceraldehyde-3-phosphate dehydrogenase in soybean at an early stage of flooding. (**A**) Gene expression of aconitase 1 (R1) in soybean at an early stage of flooding; (**B**) Gene expression of 2-oxoglutarate dehydrogenase (R3) in soybean at an early stage of flooding; (**C**) Gene expression of glyceraldehyde-3-phosphate dehydrogenase (R28) in soybean at an early stage of flooding. Two-day-old wild-type soybean plants were treated without or with flooding for one day. For ABA treatment, 10 μM ABA was supplied to wild-type soybean exposed to flooding at the same time. Two-day-old flood-tolerant mutant plants were exposed to flooding for one day. Unstressed wild-type soybean plants served as controls. Root samples (including the hypocotyl) were collected for mRNA extraction. qRT-PCR was performed and gene expression was normalized against 18S rRNA. Data shown are means ± SD from three independent biological replications. Different letters indicate statistically significant difference (*p* < 0.05, one-way ANOVA followed by Tukey’s multiple comparison test). Abbreviations are as follows: 2-OGDH, 2-oxoglutarate dehydrogenase; GAPDH, glyceraldehyde-3-phosphate dehydrogenase.

To validate the above results, protein abundance and gene expression in soybeans at an early stage of flooding were examined (Figure 6 and Figure 7). Protein abundance of R1 and R28 was increased during the development (Figure 6). Compared to flooded wild-type soybeans, protein abundance of R1, R3, and R28 was increased in ABA-treated soybeans and the flood-tolerant mutant plants (Figure 6). During development, the transcript levels of R1, R28, and R3 did not change (Figure 7). Compared with unstressed plants, flood-tolerant materials under flooding conditions showed upregulated transcript level of the gene encoding aconitase 1 (Figure 7). The transcript level of glyceraldehyde-3-phosphate dehydrogenase (GAPDH) was upregulated in flood-tolerant plants compared with flooded wild-type ones (Figure 7). Similar levels were displayed in ABA-treated soybeans and flood-tolerant mutant plants compared with unstressed plants (Figure 7). Products of quantitative reverse transcription-polymerase chain reaction (qRT-PCR) were examined and the sizes were as expected (Figure S6).

Attempts at genetic modification were utilized to achieve a desired outcome, taking advantage of correspondence with gene expression and protein abundance [23]. To screen the factors related to flood-tolerance mechanisms pointed out by our computational genetic modification effectiveness analysis, the consistency between transcript level and protein level was examined. Protein levels of aconitase 1, 2-oxoglutarate dehydrogenase, and GAPDH were lower in flooded wild-type soybeans than in unstressed plants; however, levels were higher in flood-tolerant materials than in flooded wild-type soybeans (Figure 6). Transcript levels of GAPDH did not differ between unstressed plants and flood-tolerant materials, in which higher protein levels of GAPDH were present than in flooded wild-type soybeans (Figure 6 and Figure 7).

## 3. Discussion

### 3.1. Glyceraldehyde-3-Phosphate Dehydrogenase in Glycolysis Mediate Flooding Tolerance in Soybeans

An imbalance of carbohydrate metabolism injured the flooded soybean [24]. Sugar metabolism played a role in flooding tolerance via the regulation of hexokinase and phosphofructokinase at initial stress [5]. Moreover, the balance of glycolysis was an important issue to promote flooding tolerance during the survival stages via the regulation of enolase [15]. Sucrose accumulation and inhibition of sucrose synthase induction occurred in flooded wild-type soybean [24]. Sucrose content was reduced; however, metabolite of fructose increased in flood-tolerant materials [25]. In the present study, glycolysis, fermentation, and tricarboxylic acid cycle were responsible for flooding tolerance (Figure 2). Smaller metabolite accumulation reduction ratios were obtained with modifications of GAPDH (Figure 3). Modification of GAPDH was indicated to reduce metabolite accumulation under flooding conditions (Figure 5). Taken together, these findings suggest that fructose accumulation and glycolysis are associated with flooding tolerance. In addition, GAPDH might play a role in glycolysis in response to flooding tolerance of soybeans.

The glycolytic enzyme GAPDH reversibly catalyzes the oxidation and phosphorylation of glyceraldehyde-3-phosphate to form 1,3-bisphosphoglycerate [26]. An increased activity of GAPDH mediated the glycolysis rate during adaptation to various stresses [27]. In arabidopsis, GAPDH was shown to be upregulated under anaerobic conditions, suggesting that it was linked to cellular metabolic activity [28]. 22 GAPDH genes were shown to play a role in abiotic stress tolerance in wheat [29]. Overexpression of the mushroom GAPDH in potatoes increased its salt tolerance [30]. Moreover, the loss-of-function GAPDH mutant displayed arrested root development and drastic changes in primary metabolism [31]. As shown in this study, protein levels of GAPDH were higher in flood-tolerant materials than in flooded wild-type soybean, and its transcript level did not differ significantly between flood-tolerant materials and unstressed soybean plants (Figure 6 and Figure 7). Taken together, these findings suggest that GAPDH may play a role in the flooding tolerance of soybeans through regulating the glycolytic pathway.

### 3.2. Aconitase 1 and 2-Oxoglutarate Dehydrogenase in Tricarboxylic Acid Cycle Regulate Flooding Tolerance in Soybeans

Accumulation of pyruvate was increased in flooded soybeans [21] and activation of fermentation was an important factor for acquisition of flood tolerance [13]. In this study, tricarboxylic acid cycle was another metabolic process which was markedly suppressed in wild-type soybean while slightly inhibited in flood-tolerant materials in response to flooding conditions (Figure 2). Modifications of enzymes, including aconitase 1 and 2-oxoglutarate dehydrogenase, were effective to decrease metabolite accumulation in flooded soybeans (Figure 3 and Figure 5).

Mitochondrial proteins, including aconitase and 2-oxoglutarate dehydrogenase, decreased under oxidative stress, and these reductions imposed flux restrictions on tricarboxylic acid cycle and electron transport [32]. Oxidative stress significantly affected plant mitochondria [33] and caused damage to soybeans under flooding due to oxidation and peroxide scavenging [34]. Aconitase is a central metabolic enzyme connected to mitochondrial respiration, oxidative stress responses, and cell-death regulation [35]. Under hypoxic conditions, citrate metabolism was disrupted and shifted to amino acid biosynthesis as a result of aconitase inhibition caused by nitric oxide [36]. In tobacco, aconitase was reported as an iron-regulatory protein and its disruption led to altered iron homeostasis [37]. In the present study, modifications of aconitase 1 and 2-oxoglutarate dehydrogenase declined the amounts of metabolites under flooding conditions (Figure 5); concurrently, these modifications presented with decreased protein levels in flooded wild-type soybeans but increased in flood-tolerant materials compared with unstressed soybean plants (Figure 4 and Figure 6). Taken together, these results suggest that aconitase plays a role in adenosine triphosphate synthesis and iron homeostasis; an increased protein level of aconitase might be essential to maintain flux balance in tricarboxylic acid cycle in flood-tolerant soybean plants.

2-oxoglutarate dehydrogenase is responsible for energy production and the tricarboxylic acid cycle was restored by compensatory action of gamma-aminobutyric acid shunt upon the 2-oxoglutarate dehydrogenase block [38]. Gamma-aminobutyric acid accumulated in soybeans under flooding conditions [21,33], indicating that the carbon–nitrogen ratio was modulated by the gamma-aminobutyric acid shunt pathway. Moreover, 2-oxoglutarate dehydrogenase, which was decreased under stress conditions, was shown to be an early target of oxidative stress [32]. Inhibition of 2-oxoglutarate dehydrogenase reduced the rate of respiration, coupled to changes in levels of intermediates of tricarboxylic acid cycle and amino acids crucial to nitrate assimilation [39]. As shown in this study, the protein level of 2-oxoglutarate dehydrogenase increased in flood-tolerant materials compared with flooded wild-type soybean plants (Figure 4 and Figure 6). Overall, these findings indicate that 2-oxoglutarate dehydrogenase is a pivotal enzyme governing the balance of carbon–nitrogen ratio; concurrently, an increased level of 2-oxoglutarate dehydrogenase may direct the flux into carbohydrate metabolism.

### 3.3. Proteomic Analysis and Metabolic Models as a Complementary Strategy to Screen Flood-Tolerance Factors in Glycolysis

Transcript levels and protein levels were integrated to validate flood-tolerance factors related to glycolysis and tricarboxylic acid cycle, which were highlighted by metabolic simulation. On the one hand, protein levels of glycolytic enzymes, including GAPDH, increased in flood-tolerant materials compared with flooded wild-type soybean plants; notably, their transcript levels were similar to those in unstressed plants (Figure 6 and Figure 7). On the other hand, tricarboxylic acid cycle-related proteins showed different accumulation patterns in soybeans under flooding conditions (Figure 6 and Figure 7). Tricarboxylic acid cycle is important for energy provision and has a wide range of physiological functions, such as providing essential precursor metabolites for the biosynthesis of amino acids and cellular components [40,41]. In addition, aconitase 1 and 2-oxoglutarate dehydrogenase were shown to regulate the flux balance of carbon–nitrogen ratio in plants [36,39]. Modifications of the above enzymes related to glycolysis and tricarboxylic acid cycle were examined as a comparable effect to reduce metabolite accumulation under flooding conditions; however, the glycolytic enzymes displayed a correspondence with transcript levels and protein levels. These results suggest that the discrepancy among gene expression, protein abundance, and metabolic model might be a result of the complicated flux balance between tricarboxylic acid cycle and amino acid metabolism. Collectively, the complementary strategy of protein profiling and metabolic modeling might have potential to screen flood-tolerant proteins in the glycolytic pathway in soybeans.

## 4. Materials and Methods

### 4.1. Plant Materials and Treatments

Soybean seeds of both the wild type (*Glycine max* L. (Merrill) cultivar Enrei) and flood tolerant were collected from the National Institute of Crop Science, Japan. The seeds were surface sterilized using 3% sodium hypochlorite solution, rinsed in water, and then sown in 450 mL of silica sand with water in a seedling case (150 mm × 60 mm × 100 mm). The flood-tolerant soybeans, whose root growth of M6 stage was not suppressed even under flooding conditions, were generated by gamma-ray irradiation and confirmed by flood-tolerant tests [13]. Two-day-old wild-type soybean plants were treated with and without flooding by adding 700 mL of extra water for 1, 2, 3, and 4 days. Wild-type soybean plants were exposed to flooding with 10 μM ABA (MP Biomedicals, Santa Ana, CA, USA) at the same time [14]. Two-day-old flood-tolerant mutant plants were exposed to flooding for 1, 2, 3, and 4 days. Soybean plants were grown in a growth chamber illuminated with white fluorescent light (160 µmol m^−2^ s^−1^, 16 h/8 h dark photoperiod) at 25 °C. Unstressed wild-type soybean served as controls. For sampling, cotyledon and 5 mm length from the end of hypocotyl were excluded; however, the remaining parts of the plant were collected as the section of seedling length, which consists of the root including the hypocotyl (Figure S1). Three independent experiments were performed as biological replications for morphological, proteomic, and gene expression analyses. Biological replications consisted of soybean plants sown on different days and 10 plants were sampled at each time point for each treatment.

### 4.2. Extraction of Proteins

A portion (0.5 g) of the root (including the hypocotyl) was ground to a fine powder in liquid nitrogen with a mortar and pestle. The powder was added to an extraction buffer consisting of 10% (*v/v*) trichloroacetic acid and 0.07% (*v/v*) 2-mercaptoethanol in acetone, and the resulting suspension was vortexed thoroughly. The mixture was sonicated for 10 min, incubated for 60 min at −20 °C, and vortexed every 15 min. The suspension was centrifuged at 9000× *g* for 20 min at 4 °C; supernatant was discarded and pellet was washed twice with 0.07% (*v/v*) 2-mercaptoethanol in acetone. The pellet was dried using a Speed-Vac concentrator (Savant Instruments, Hicksville, NY, USA) and re-suspended in lysis buffer containing 8 M urea, 2 M thiourea, 5% CHAPS, and 2 mM tributylphosphine by vigorous vortexing for 60 min [42]. The suspension was centrifuged at 20,000× *g* for 20 min and supernatant was collected as protein extract. The protein concentration was determined using the Bradford method [43] with bovine serum albumin (Sigma-Aldrich, St. Louis, MO, USA) as the standard.

### 4.3. Enrichment and Digestion of Proteins

Proteins (150 μg) in lysis buffer were enriched using methanol and chloroform to remove the detergent [44]. Methanol (600 μL) was added to 150 μL of the protein sample and the solution was thoroughly mixed before adding 150 μL of chloroform. After vortexing, 450 μL water was added to induce phase separation and the solution was centrifuged at 20,000× *g* for 10 min. The upper phase was discarded and 450 μL of methanol was added. The sample was further centrifuged at 20,000× *g* for 10 min, supernatant was discarded, and pellet was dried. The dried pellet was re-suspended in 50 mM NH_4_HCO_3_, reduced with 50 mM dithiothreitol for 30 min at 56 °C, and then alkylated with 50 mM idoacetamide for 30 min at 37 °C in the dark. Alkylated proteins were digested with trypsin (Wako, Osaka, Japan) at a ratio of 1:100 enzyme/protein at 37 °C for 16 h. The peptides were acidified with formic acid (pH < 3) and centrifuged at 20,000× *g* for 10 min. The supernatant was collected and peptides were analyzed by nanoliquid chromatography (LC)-mass spectrometry (MS)/MS.

### 4.4. Mass Spectrometry Analysis

Each peptide sample was separated using an Ultimate 3000 nanoLC system (Dionex, Germering, Germany) and peptide ions were detected using a nanospray LTQ Orbitrap Discovery MS (Thermo Fisher Scientific, Waltham, MA, USA) in data-dependent acquisition mode with installed Xcalibur software (version 2.0.7; Thermo Fisher Scientific). Peptides (4 μL) were loaded onto a C18 PepMap trap column (300 µm ID × 5 mm; Dionex) equilibrated with 0.1% (*v/v*) formic acid and eluted with a linear acetonitrile gradient (8–30% over 150 min) in 0.1% (*v/v*) formic acid at a flow rate of 200 nL/min. The eluted peptides were separated on a C18 capillary tip column (75 µm ID × 120 mm; Nikkyo Technos, Tokyo, Japan) with a spray voltage of 1.5 kV. Full-scan mass spectra were acquired in the MS range of 400 to 1500 m/z with a resolution of 30,000. The lock mass function was used to obtain high mass accuracy [45], and the ions C_24_H_39_O_4_^+^ (*m*/*z* 391.28429), C_14_H_46_NO_7_Si_7_^+^ (*m*/*z* 536.16536) and C_16_H_52_NO_8_Si_8_^+^ (*m*/*z* 610.18416) were used. The ten most intense precursor ions were selected for collision-induced fragmentation in linear ion trap at a normalized collision energy of 35%. Dynamic exclusion was employed within 90 s to prevent repetitive selection of peptides [46]. The acquired MS spectra were used to identify proteins.

### 4.5. Protein Identification from Acquired Mass Spectrometry Data

Proteins were identified using Mascot search engine (version 2.5.1; Matrix Science, London, UK) and Proteome Discoverer software (version 1.4.0.288; Thermo Fisher Scientific) against Phytozome-*Glycine max* peptide database (Phytozome version 12.0, https://phytozome.jgi.doe.gov/) [47]. The following parameters were used for Mascot search: carbamidomethylation of cysteine was set as fixed modification; oxidation of methionine was set as variable modification; trypsin was used as specific proteolytic enzyme; one missed cleavage was allowed; peptide mass tolerance was 10 ppm; fragment mass tolerance was 0.8 Da; and peptide charges were +2, +3, and +4. The peptide cut-off score was 10 and the S/N threshold (FT-only) was 1.5 for peak filtration. An automatic decoy database search was performed as part of the search. Mascot Percolator was used to improve the accuracy and sensitivity of peptide identification [48]. False discovery rates (false positive/(false postive+true positive)) for peptide identification were less than 0.01. Peptides with a percolator ion score of more than 13 (*p* < 0.05) were used for protein identification. The search results were exported as XML format for comparative analysis.

### 4.6. Analysis of Differential Protein Abundance

Relative abundance of peptides and proteins under different experimental conditions was compared using commercial label-free quantification package SIEVE software (version 2.1.377; Thermo Fisher Scientific). Chromatographic peaks detected by MS were aligned, and peptide peaks were detected as a frame on all parent ions scanned by MS/MS using 5 min frame time width and 10 ppm frame *m*/*z* width. The area of chromatographic peak within a frame was compared for each sample and ratios between samples were determined for each frame. The frames with MS/MS scan were matched to Mascot search results. The ratios between samples were determined from variance-weighted average of ratios in frames, which matched the peptides in the MS/MS spectrum. The ratios of peptides were further integrated to determine the ratios of corresponding proteins. Total ion current was used for normalization in a differential analysis of protein abundance. The outliers of ratio were deleted in the frame table filter based on frame area. Changes of protein abundance between samples were considered significant at *p* < 0.05. The minimum requirement for protein identification was two significant matched peptides.

### 4.7. Functional Categorization

Functional categorization was performed using MapMan bin codes [49]. Protein level was visualized using MapMan software (version 3.6.0RC1) [50]. The software and mapping file (Gmax_109_peptide) were downloaded from MapMan website (http://mapman.gabipd.org/web/guest/mapman).

### 4.8. Cluster Analysis of Protein Level

Cluster analysis of protein level was conducted using Genesis software (version 17.6, http://genome.tugraz.at) [51].

### 4.9. Metabolic Network Modeling

Temporal profiles of metabolites in soybeans under flooding and control conditions were reported by Nakamura et al. [21], forming the basis on which to construct an equation model of the soybean metabolic system, which included glycolysis, fermentation, tricarboxylic acid cycle, and gamma-aminobutyric acid shunt [20]. In the model, the Michaelis constants (*K*_m_ values, Table S5) were set to the values listed in the BRENDA database [52]. If there were multiple *K*_m_ values, the value for the enzyme in the species most closely related to soybeans and in conditions most closely related to flooding was selected, as described by Sakata et al. [20]. Modification of an enzyme was mimicked by changing the *V*_max_ value in the corresponding reaction model. Overexpression or suppression of the enzyme was mimicked by increasing or decreasing, respectively, the *V_max_* value from its initial value (Table S5). After adjustment of *V*_max_ value in each reaction, the simulated amounts of fitting-target metabolites were fitted to the experimental metabolic data of flooded soybean documented by Nakamura et al. [21]. The log-transformed simulated data and experimental data of the fitting-target metabolites were plotted against each other. A coefficient of determination (*r*^2^) value of 0.95 was considered to indicate that the model predication was in agreement with the experimental data. Metabolite accumulation reduction ratio, which was the amount of metabolite in the modified case divided by the amount in the unmodified case, was calculated by using the model after fitting. The effect of modification was considered by subtracting the average accumulation reduction ratio from one. The whole calculation model was constructed on the metabolic pathway calculation program Winbest-kit [53] and the complete model in Winbest-kit code is available upon request to the corresponding authors.

### 4.10. RNA Extraction and Quantitative Reverse Transcription-Polymerase Chain Reaction

A portion (0.1 g) of the root (including the hypocotyl) was ground to a fine powder in liquid nitrogen using a sterilized mortar and pestle. Total RNA was extracted using the RNeasy Plant Mini Kit (Qiagen, Hilden, Germany), treated with DNase I (Qiagen), and then reverse transcribed into cDNA using iScript Reverse Transcription Supermix (Bio-Rad, Hercules, CA, USA) according to manufacturer’s instructions. qRT-PCR was performed in the reaction mixture (10 μL) using SsoAdvanced SYBR Green Supermix (Bio-Rad) on a MyiQ Single-Color Real-Time PCR Detection system (Bio-Rad). The PCR cycling conditions were as follows: 95 °C for 30 s, 45 cycles of 95 °C for 10 s, and 60 °C for 30 s. The transcript levels were normalized against 18S rRNA (X02623.1). Three biological replications were analyzed and each biological replication was technically duplicated to reduce error rate. qRT-PCR primers were designed using Primer3Plus (http://www.bioinformatics.nl/cgi-bin/primer3plus/primer3plus.cgi/) (Table S6). Primer specificity was confirmed by BLASTN searches against Phytozome-*Glycine max* database with the designed primers as well as a melting curve analysis. The amplified fragments were separated by 2% agarose gel electrophoresis.

### 4.11. Statistical Analysis

Student’s *t*-test was used for comparisons between two groups and it was performed using GraphPad Prism 6 (version 6.03; GraphPad Software; La Jolla, CA, USA). One-way ANOVA, followed by Tukey’s multiple comparison, was used for comparison among multiple groups and it was conducted using SPSS (version 22.0; IBM, Armonk, NY, USA). A *p* < 0.05 was considered as statistically significant.

### 4.12. Accession Codes

Mass spectrometry proteomics data have been deposited to the ProteomeXchange Consortium [54] via the PRIDE partner repository with the dataset identifier PXD005680. The metabolomic experimental data have been deposited in the following web site: “proteome.dc.affrc.go.jp/Soybean/metabo/metabolism_tbl.html”.

## 5. Conclusions

**Figure 8:**
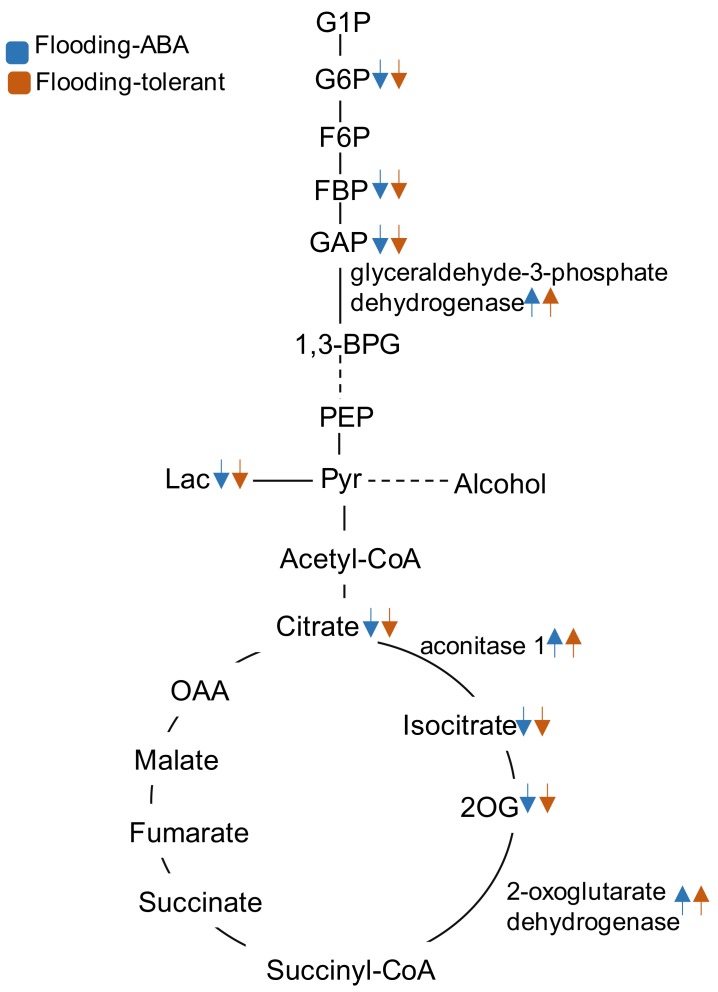
Schematic summary of major physiological processes in soybean roots under flooding conditions. On the basis of proteomic data, glycolysis, fermentation, and tricarboxylic acid cycle were highlighted as the major metabolisms affected in soybean roots under flooding. Aconitase 1,2-oxoglutarate dehydrogenase, and glyceraldehyde-3-phosphate dehydrogenase configured the candidates for flood-tolerance mechanisms. Metabolic simulation was conducted by concurrent modification of *V*_max_ values of these three enzymes. Blue and orange colors present flooded soybean with ABA treatment and flood-tolerant plant, respectively. Protein abundance of aconitase 1,2-oxoglutarate dehydrogenase, and glyceraldehyde-3-phosphate dehydrogenase and metabolite accumulation predicated by metabolic simulation were presented in the ABA-treated soybean and flood-tolerant plant. Upward and downward arrows indicate increase and decrease, respectively, in protein abundance or metabolite accumulation in ABA-treated soybean and flood-tolerant plant compared to wide-type soybean under flooding. The abbreviations of metabolites are the same as described in the legend of Figure 3.

Previous studies have attempted to uncover flood-tolerant mechanisms in soybeans using proteomic [15], transcriptomic [55], and metabolomic [5] techniques. The metabolic model was developed for soybeans [20] based on metabolite profiles under flooding conditions [21]. Consistent with previous results, metabolisms of glycolysis and tricarboxylic acid cycle were responsible for flooding stress [5,7] The balance of glycolysis and activation of fermentation were especially critical for flooding tolerance in soybeans [13,15]. Additional findings in the present study were as follows (Figure 8): (i) flood-tolerant materials, including a mutant and ABA-treated soybean, grew better than wild-type plants exposed to flooding; (ii) glycolysis and tricarboxylic acid cycle were enhanced in flood-tolerant materials compared with flooded wild-type soybean plants; (iii) protein levels of GAPDH, aconitase 1, and 2-oxoglutarate dehydrogenase were higher in flood-tolerant materials than in flooded wild-type soybean plants; (iv) metabolic simulations of GAPDH, aconitase 1, and 2-oxoglutarate dehydrogenase led to a reduction in metabolite accumulation under flooding; and (v) transcript levels of GAPDH were similar in flood-tolerant materials and in unstressed plants. Altogether, the present study indicates that energy provision and flux balance of carbon–nitrogen might play a role in soybean flooding tolerance. In addition, a proteomic approach combined with computational genetic modification-effectiveness analysis was useful to screen flood-tolerant enzymes in glycolytic pathway in soybeans.

## Figures and Tables

**Table 1:** Ratio of proteins related to glycolysis, fermentation, and tricarboxylic acid cycle in wild-type soybean plants under flooding conditions.

	Reaction	Protein ID	Description	Ratio (Control Plant)					Ratio (Flooded Wile-Type Soybean)				Functional Category
				2-day-old	3-day-old	4-day-old	5-day-old	6-day-old	1-day-flooded	2-day-flooded	3-day-flooded	4-day-flooded	
1	R1a/b	Glyma11g08550.1	aconitase 1	1	1.26	2.01	3.88	11.61	0.88	1.00	1.27	1.34	TCA cycle
2	R2	Glyma10g06590.1	isocitrate dehydrogenase V	1	1.00	1.00	1.00	1.00	0.96	0.77	0.71	0.51	TCA cycle
3	R3a/b	Glyma18g52430.2	2-oxoglutarate dehydrogenase	1	1.00	1.00	1.00	1.00	0.82	0.83	0.90	1.16	TCA cycle
4	R5a/b	Glyma11g07250.1	succinate dehydrogenase 1-1	1	0.72	0.49	0.71	1.70	0.86	1.00	0.81	0.74	TCA cycle
5	R7a/b	Glyma06g34190.1	malate dehydrogenase	1	6.11	1.61	8.88	19.44	1.51	6.55	3.48	4.28	TCA cycle
6	R8c	Glyma15g42301.1	citrate synthase family protein	1	1.00	1.00	1.00	1.00	1.00	1.00	1.00	1.00	TCA cycle
7	R11a/b	Glyma06g12780.1	alcohol dehydrogenase 1	1	1.00	1.00	1.00	1.00	2.99	4.20	6.21	22.61	fermentation
8	R14	Glyma05g09310.2	pyruvate kinase family protein	1	2.74	2.36	1.19	5.66	1.06	1.29	1.46	1.47	glycolysis
9	R18	Glyma13g36670.1	phosphoenolpyruvate carboxylase 1	1	2.12	1.45	1.26	2.98	0.96	1.09	0.86	1.20	glycolysis
10	R25a/b	Glyma19g37520.1	enolase	1	1.00	1.00	1.00	1.00	1.16	1.53	2.49	3.25	glycolysis
11	R26a/b	Glyma18g45121.1	phosphoglycerate mutase	1	1.00	1.00	1.00	1.00	1.03	1.12	1.35	0.79	glycolysis
12	R27a/b	Glyma15g41550.1	phosphoglycerate kinase	1	1.00	1.00	1.00	1.00	0.91	1.14	1.16	4.82	glycolysis
13	R28a/b	Glyma04g36860.1	glyceraldehyde-3-phosphate dehydrogenase	1	3.30	1.92	2.23	23.93	1.21	1.41	2.14	4.08	glycolysis
14	R29a/b	Glyma03g34950.1	aldolase superfamily protein	1	1.00	1.00	1.00	1.00	1.00	1.00	1.00	1.00	glycolysis
15	R32a/b	Glyma20g32030.1	phosphoglucomutase	1	0.05	0.02	0.06	0.12	0.64	1.07	0.91	0.25	glycolysis

Reaction, according to the simulation program; Protein ID, according to Phytozome soybean genome database; Ratio, relative abundance of protein; Functional category, protein function categorized using MapMan bin codes; TCA, tricarboxylic acid. Ratio indicates protein abundance at each time point compared with 2-day-old unstressed wild-type soybean plants.

**Table 2:** Ratio of commonly changed proteins related to glycolysis, fermentation, and tricarboxylic acid cycle in ABA-treated wild-type soybeans and flood-tolerant mutant plants under flooding conditions.

	Reaction	Protein ID	Description	Ratio (Control Plant)	Ratio (Flooded Wile-Type Soybean with ABA Treatment)				Ratio (Flooded soybean of flooding-Tolerant Mutant)				Functional Category
				2-day-old	1-day-flooded	2-day-flooded	3-day-flooded	4-day-flooded	1-day-flooded	2-day-flooded	3-day-flooded	4-day-flooded	
1	R1a/b	Glyma11g08550.1	aconitase 1	1	3.07	4.97	19.17	2.45	2.17	2.31	8.04	1.95	TCA cycle
2	R2	Glyma14g39160.2	cytosolic NADP dependent isocitrate dehydrogenase	1	8.37	28.68	6.89	5.77	1.30	1.44	4.86	1.60	TCA cycle
3	R3a/b	Glyma18g52430.2	2-oxoglutarate dehydrogenase	1	1.35	2.00	3.75	0.91	1.01	1.53	2.52	1.03	TCA cycle
4	R5a/b	Glyma01g38200.1	succinate dehydrogenase 1-1	1	0.97	0.18	1.04	0.53	0.70	0.39	0.28	0.78	TCA cycle
5	R7a/b	Glyma06g34190.1	malate dehydrogenase	1	3.84	3.88	2.62	1.28	5.32	10.52	11.90	17.32	TCA cycle
6	R8a/b	Glyma09g04000.2	ATP citrate lyase B1	1	9.24	8.11	5.52	2.51	1.42	1.50	4.25	1.37	TCA cycle
7	R11a/b	Glyma09g08150.1	aldehyde dehydrogenase 7B4	1	12.26	16.00	3.60	1.69	1.19	1.28	7.70	1.16	fermentation
8	R14	Glyma05g09310.2	pyruvate kinase family protein	1	9.79	7.88	10.14	10.41	1.71	1.93	1.75	1.91	glycolysis
9	R18	Glyma06g33380.1	phosphoenolpyruvate carboxylase 3	1	2.90	11.44	3.38	1.17	1.19	0.93	3.86	0.97	glycolysis
10	R25a/b	Glyma03g34830.1	enolase	1	7.34	14.18	14.10	3.11	9.18	3.92	8.87	2.89	glycolysis
11	R26a/b	Glyma09g40690.1	phosphoglycerate mutase 2,3 bisphosphoglycerate	1	9.98	12.68	19.37	1.65	1.42	1.72	3.35	1.37	glycolysis
12	R27a/b	Glyma08g17600.1	phosphoglycerate kinase	1	7.29	5.40	18.41	1.50	1.26	1.54	10.29	1.49	glycolysis
13	R28a/b	Glyma04g36860.1	glyceraldehyde-3-phosphate dehydrogenase	1	5.96	19.08	20.78	3.83	2.10	2.31	7.22	2.23	glycolysis
14	R29a/b	Glyma03g34950.1	aldolase superfamily protein	1	3.70	8.76	8.26	2.42	2.05	2.16	3.99	1.71	glycolysis
15	R32a/b	Glyma05g34790.1	phosphoglucomutase	1	3.97	19.52	11.35	0.86	2.69	3.70	7.08	5.45	glycolysis

Reaction, according to the simulation program; Protein ID, according to Phytozome soybean genome database; Ratio, relative abundance of protein; Functional category, protein function categorized using MapMan bin codes, tricarboxylic acid. Ratio indicates protein abundance at each time point compared with 2-day-old unstressed wild-type soybean plants. Proteins, which presented with same change tendency of abundance in ABA-treated soybeans and flooding-tolerant mutant plants, were selected.

## Supplementary Materials

Supplementary materials can be found at http://www.mdpi.com/1422-0067/19/5/1301/s1.

## Abbreviations

ABA	Abscisic Acid
LC	Liquid Chromatography
GAPDH	Glyceraldehyde-3-Phosphate Dehydrogenase
MS	Mass Spectrometry
qRT-PCR	Quantitative Reverse Transcription-Polymerase Chain Reaction
