# Supplementary material for: An Integrated Approach of Proteomics and Computational Genetic Modification Effectiveness Analysis to Uncover the Mechanisms of Flood Tolerance in Soybeans
Source: Int J Mol Sci. 2018 Apr 26;19(5):1301. doi: 10.3390/ijms19051301 (PMC5983631; doi:10.3390/ijms19051301)
Supplement: Supplementary file 1 [file ijms-19-01301-s001.zip › ijms-285358-supp-final/Figures S1-S6.pdf]

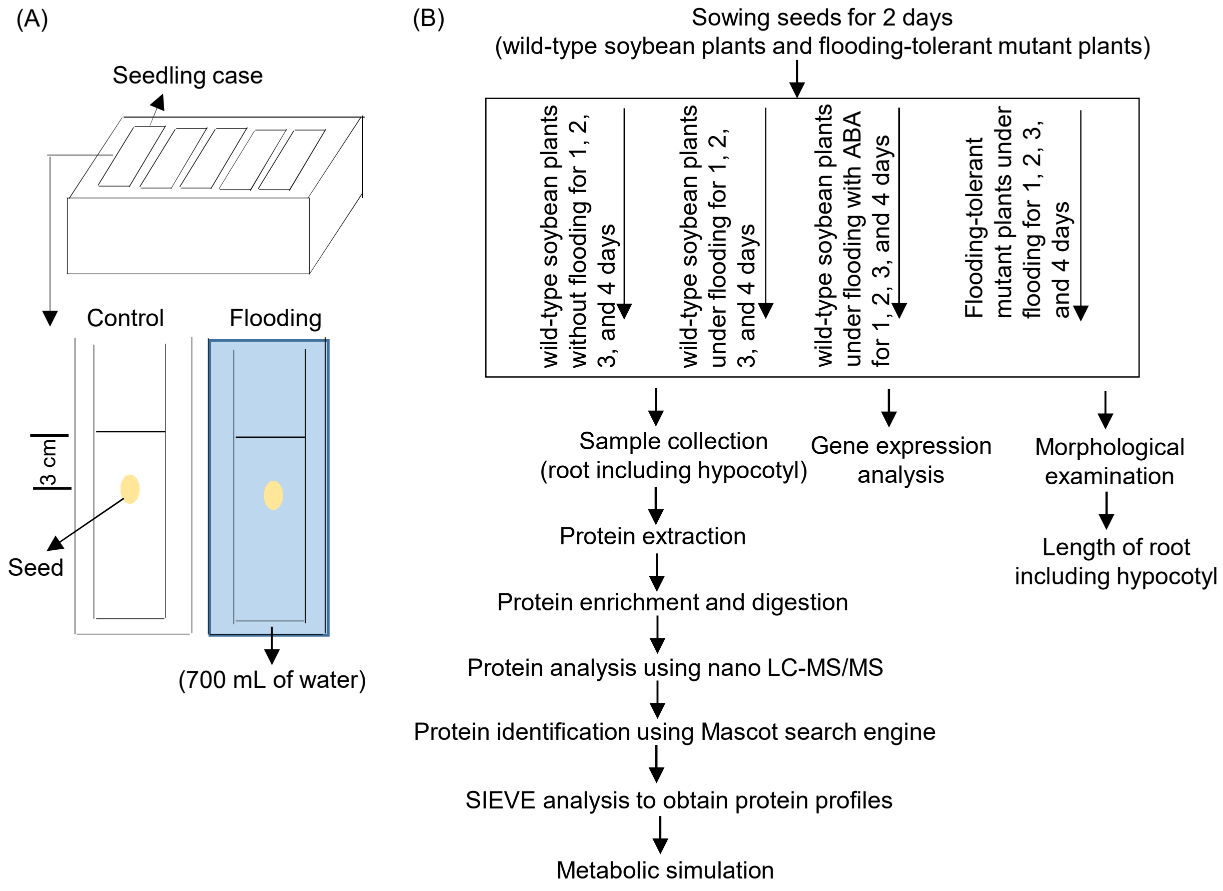

Figure S1. Experimental design of present study. (A) Flooding stress conducted in present study. (B) Experiments performed in present study. Two-day-old wild-type soybean plants were treated without or with flooding by adding 700 mL of extra water for 1, 2, 3, and 4 days. For ABA treatment, 10 μM ABA was supplied to wild-type soybean plants exposed to flooding at the same time. Two-day-old flooding-tolerant mutant plants were exposed to flooding for 1, 2, 3, and 4 days. Unstressed wild-type soybean plants were served as controls. Plant morphology was examined at each time point. Samples of root (including hypocotyl) were collected for proteomic and gene expression analyses. Proteins related to predominant metabolisms affected by flooding were subjected to metabolic simulation. Gene expression was examined to validate proteomic and simulation results.

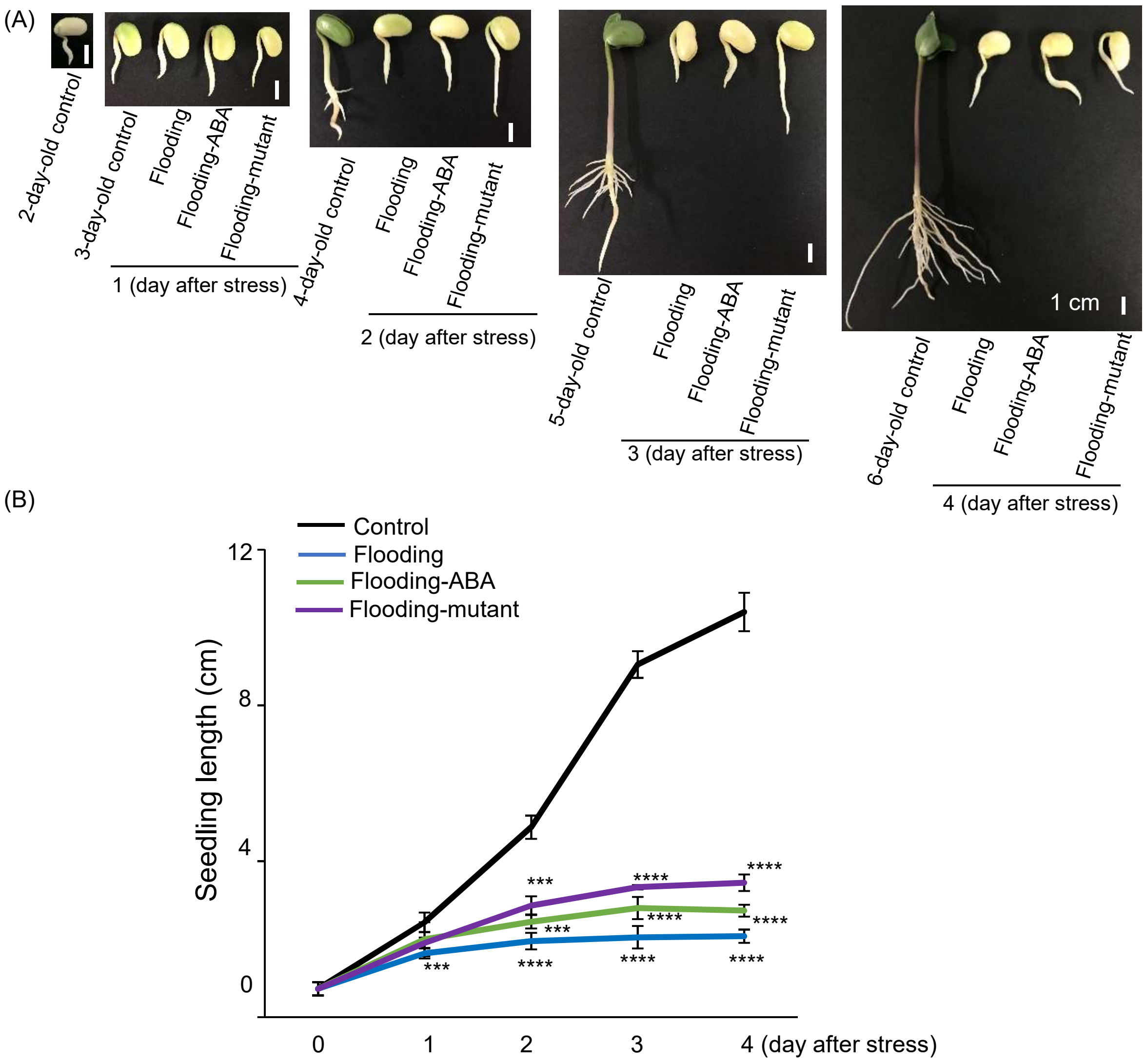

Figure S2. Morphology and seedling length of wild-type soybean, ABA-treated soybean, and flooding-tolerant mutant soybean plants under flooding. (A) Morphology of wild-type soybean, ABA-treated soybean and flooding-tolerant mutant soybean plants exposed to flooding conditions. (B) Seedling length of wild-type soybean, ABA-treated soybean and flooding-tolerant mutant soybean plants exposed to flooding conditions. Two-day-old wild-type soybean plants and flooding-tolerant mutant plants were treated with flooding for 1, 2, 3, and 4 days. For ABA treatment, 10 μM ABA was supplied to wild-type soybean plants exposed to flooding at the same time. Unstressed wild-type soybean plants were served as controls. Length of root including hypocotyl was measured at indicated time point during flooding. Photographs show soybean plants at each time point without or with flooding. Bar = 1 cm. Data are mean ± SD from three independent biological replications. At each time point, values in treatments were compared with value in unstressed control. ***p < 0.001 and ****p < 0.0001 (Student's t-test).

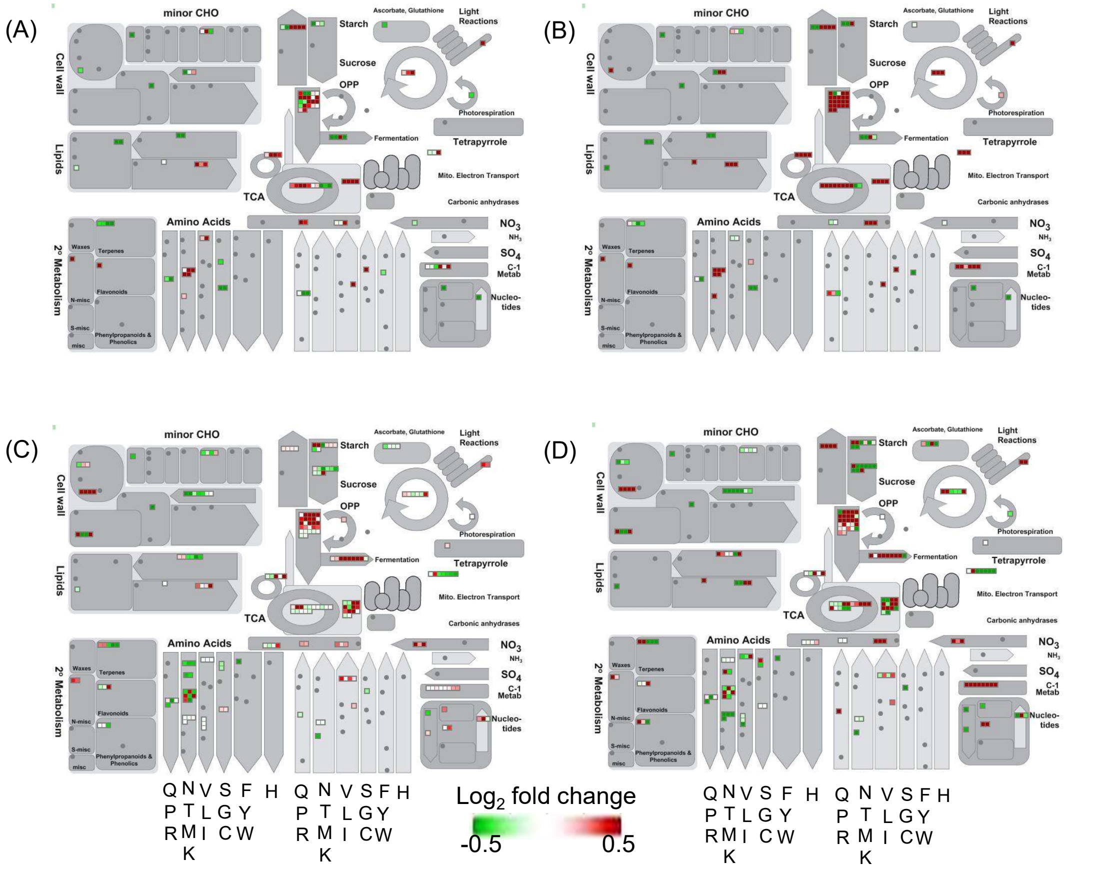

Figure S3. Abundance of proteins mapped to primary metabolism in wild-type soybean under flooding. (A) Four-day-old wild-type soybean without flooding stress. (B) Six-day-old wild-type soybean without flooding stress. (C) Four-day-old wild-type soybean flooded for 2 days. (D) Six-day-old wild-type soybean flooded for 4 days. Two-day-old wild-type soybean plants were treated without or with flooding for 2 and 4 days. Unstressed wild-type soybean plants were served as controls. Green and red colors indicate a decrease and increase, respectively, in fold change values compared with 2-day-old unstressed wild-type soybean plants. Abbreviations are as follows: Q, Glutamine; P, Proline; R, Arginine; N, Asparagine; T, Threonine, M, Methionine; K, Lysine; V, Valine; L, Leucine; I, Isoleucine; S, Serine; G, Glycine; C, Cysteine; F, Phenylalanine; Y, Tyrosine; W, Tryptophan; H, Histidine.

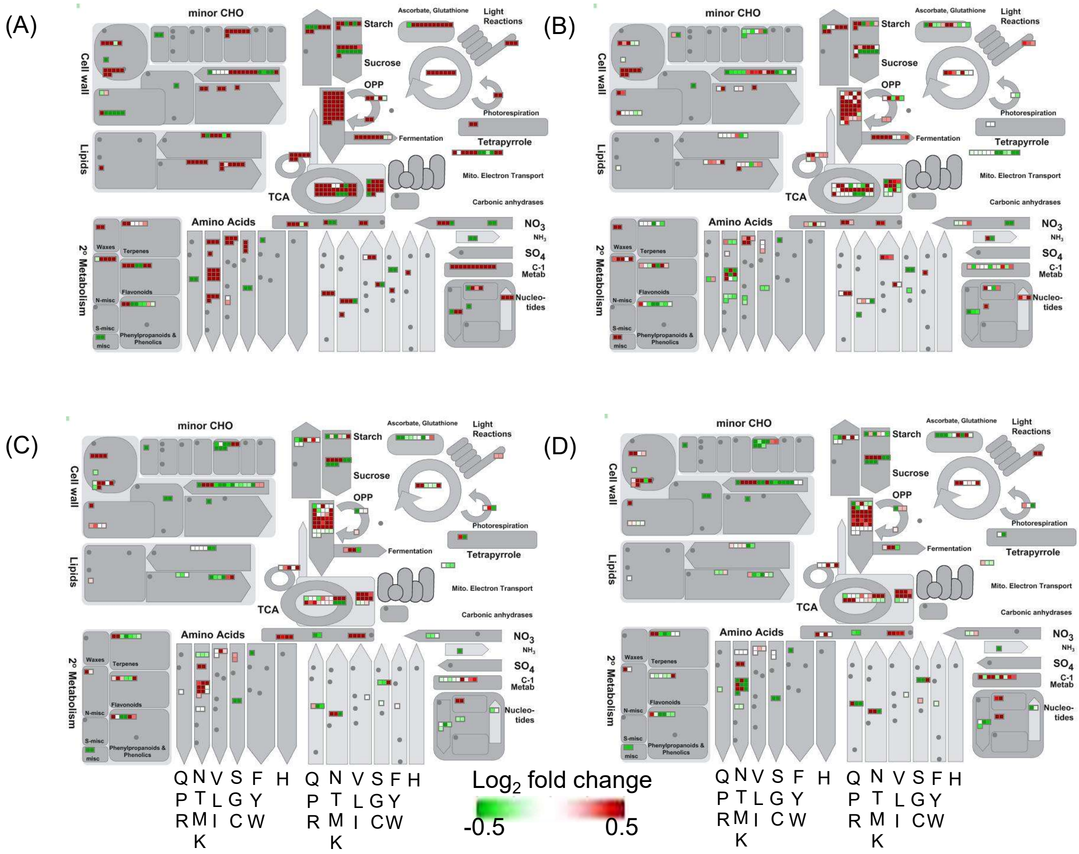

Figure S4. Abundance of proteins mapped to primary metabolism in ABA-treated soybean and flooding-tolerant mutant soybean plants under flooding. (A) Four-day-old wild-type soybean flooded for 2 days coupled with ABA treatment. (B) Six-day-old wild-type soybean flooded for 4 days coupled with ABA treatment. (C) Four-day-old flooding-tolerant soybean flooded for 2 days. (D) Six-day-old flooding-tolerant soybean flooded for 4 days. Unstressed wild-type soybean plants were served as controls. Green and red colors indicate a decrease and increase, respectively, in fold change values compared with 2-day-old unstressed wild-type soybean plants. Abbreviations are as follows: Q, Glutamine; P, Proline; R, Arginine; N, Asparagine; T, Threonine, M, Methionine; K, Lysine; V, Valine; L, Leucine; I, Isoleucine; S, Serine; G, Glycine; C, Cysteine; F, Phenylalanine; Y, Tyrosine; W, Tryptophan; H, Histidine.

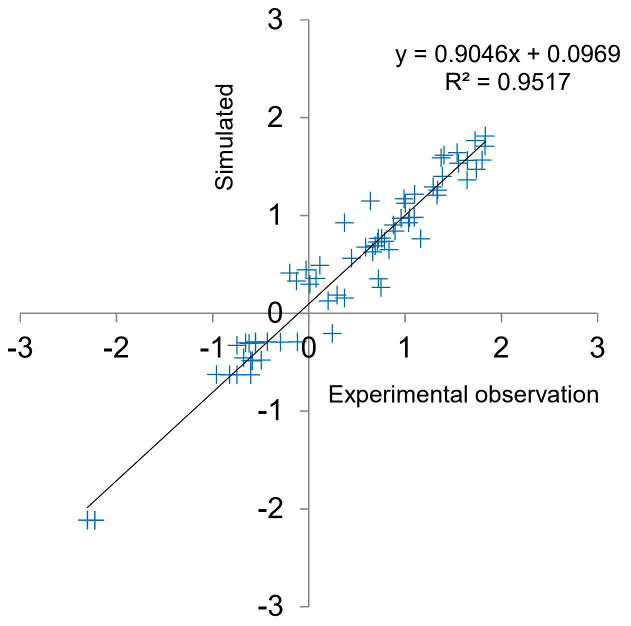

Figure S5. Log-log plot of experimental versus simulated data for fitting-target metabolites.

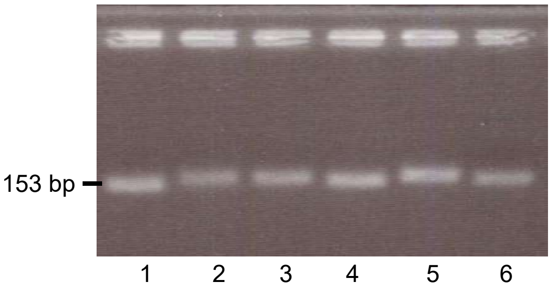

1, 18S rRNA (153 bp);
2, aconitase 1 (239 bp);
3, 2-oxoglutarate dehydrogenase (221 bp);
4, malate dehydrogenase (172 bp);
5, glyceraldehyde-3-phosphate dehydrogenase (228 bp);
6, phosphoglucomutase (181 bp).

Figure S6. Agarose gel electrophoresis of qRT-PCR products. qRT-PCR products obtained using primers listed in Table S6 were separated on 2% agarose gels and stained with ethidium bromide.
